# Apolipoprotein E Induces Lipid Accumulation Through Dgat2 That Is Prevented with Time-Restricted Feeding in *Drosophila*

**DOI:** 10.3390/genes15111376

**Published:** 2024-10-25

**Authors:** Ruan C. M. Moraes, Jonathan R. Roth, Hailey Mao, Savannah R. Crawley, Brittney P. Xu, John C. Watson, Girish C. Melkani

**Affiliations:** 1 Department of Pathology, Division of Molecular and Cellular Pathology, Heersink School of Medicine, University of Alabama at Birmingham, Birmingham, AL 35294, USA; 2 Department of Psychiatry and Behavioral Neurobiology, Heersink School of Medicine, University of Alabama at Birmingham, Birmingham, AL 35294, USA; 3 Department of Neurobiology, Heersink School of Medicine, University of Alabama at Birmingham, Birmingham, AL 35294, USA; 4 UAB Nathan Shock Center, 1300 University Boulevard, Birmingham, AL 35294, USA

**Keywords:** apolipoprotein E (ApoE), ApoE-alleles, lipid metabolism, diacylglycerol O-acyltransferase 2 (*Dgat2*), Alzheimer’s disease (AD), time-restricted feeding (TRF), drosophila model

## Abstract

**Background:** Apolipoprotein E (ApoE) is the leading genetic risk factor for late-onset Alzheimer’s disease (AD), which is the leading cause of dementia worldwide. Most people have two ApoE-ε3 (ApoE3) alleles, while ApoE-ε2 (ApoE2) is protective from AD, and ApoE-ε4 (ApoE4) confers AD risk. How these alleles modulate AD risk is not clearly defined, and ApoE’s role in lipid metabolism is also not fully known. Lipid droplets increase in AD. However, how ApoE contributes to lipid accumulation in the brain remains unknown. **Methods:** Here, we use *Drosophila* to study the effects of ApoE alleles on lipid accumulation in the brain and muscle in a cell-autonomous and non-cell-autonomous manner. **Results:** We report that pan-neuronal expression of each ApoE allele induces lipid accumulation specifically in the brain, but not in the muscle. However, this was not the case when expressed with muscle-specific drivers. ApoE2- and ApoE3-induced lipid accumulation is dependent on the expression of Dgat2, a key regulator of triacylglycerol production, while ApoE4 still induces lipid accumulation even with knock-down of Dgat2. Additionally, we find that implementation of time-restricted feeding (TRF), a dietary intervention in which food access only occurs in the active period (day), prevents ApoE-induced lipid accumulation in the brain of flies and modulates lipid metabolism genes. **Conclusions:** Altogether, our results demonstrate that ApoE induces lipid accumulation in the brain, that ApoE4 is unique in causing lipid accumulation independent of Dgat2, and that TRF prevents ApoE-induced lipid accumulation. These results support the idea that lipid metabolism is critical in AD, and that TRF could be a promising therapeutic approach to prevent ApoE-associated dysfunction in lipid metabolism.

## 1. Introduction

Alzheimer’s disease (AD) is the leading cause of neurodegeneration worldwide and is projected to grow rapidly going forward [1]. The largest genetic risk factor for AD is apolipoprotein E, with three common alleles: ε2, ε3, and ε4, (referred to as ApoE2, E3, or E4), where ApoE4 carriers are 10–20 times more likely to obtain AD than ApoE3 carriers. Although nearly three decades have passed since the link between ApoE4 and Alzheimer’s disease was discovered [2], the mechanistic link between ApoE and AD is not well understood. Apolipoproteins are responsible for transporting lipids between cells and are important for the maintenance of cognition, as knocking out ApoE results in cognitive deficits [3] in an allele-dependent manner [4]. In the brain, ApoE is mostly expressed in glia [5] but is also present in neurons [6]. Neuronal ApoE expression increases under neuronal stress and degeneration [7], and removing neuronal ApoE4 prevents AD-associated dysfunction in a mouse model of AD [8]. ApoE controls the distribution of lipids between cells and maintains lipid homeostasis, which is necessary to produce new synapses and maintenance of the fluidity of the lipid bilayer [9,10]. Additionally, ApoE is critical for neuron–glia communication and transports triacylglycerol from neurons to astrocytes, where it is degraded [11,12,13]. In recent years, research relating ApoE with the formation of lipid droplets has intensified, demonstrating that ApoE4 can increase lipid droplets and influence the composition of glycerols and fatty acids (FA) present in droplets [14]. However, it has not yet been elucidated whether this increase in the formation of lipid droplets is detrimental or protective in the context of AD.

The importance of lipids in AD was identified in Alzheimer’s initial descriptions, which noted abnormal intracellular lipid accumulation in the brain [15,16]. This finding has been built on by recent genome-wide association studies that associate polymorphisms in lipid metabolism genes with AD risk, especially ApoE [17,18]. The brain is lipid-rich, especially with myelin in white matter tracts, and disrupted myelin can on its own drive amyloid-β deposition [19], and aberrant white matter changes are seen in AD patients and with aging [20]. Lipidomic studies in preclinical models show that ApoE is critical for multiple lipid metabolism pathways and for circulating lipids [21,22,23], directly linking ApoE to lipid metabolism in the context of AD. The entorhinal cortex is specifically susceptible to ApoE4-induced decreases in diacylglycerol levels with associated increased monoacylglycerol and sphingolipids [23]. As ApoE is the most common lipoprotein in the brain [24], its clear role in regulating lipid metabolism in the brain [25] likely contributes to the AD risk associated with different ApoE alleles. ApoE also is important for neuroinflammation. In humans, ApoE4 carriers have elevated plasma C-reactive protein levels compared to age and sex-matched ApoE2 and ApoE3 carriers [26]. Additionally, ApoE has a neuroprotective role in astrocytes, controlling neuroinflammation, while ApoE4 leads to neuroinflammation [27].

One common link between AD, lipids, metabolism, aging, and ApoE is circadian rhythms. Circadian rhythms are self-sustaining, ~24 h rhythms generated by cell-autonomous transcriptional feedback loops [28,29], whose molecular mechanisms were identified in *Drosophila melanogaster* (fruit flies) and are largely conserved in mammals [30,31]. Circadian rhythms become disrupted with aging [32,33,34,35,36], AD [37,38,39,40,41,42,43], and sleep disruptions, which are common in AD patients [37,38,39,44,45,46]. The presence of ApoE4 is associated with sleep disorders, including obstructive sleep apnea/hypopnea [47], as well as increased sleep in people with cognitive impairment [48]. In addition, improved sleep decreases the predisposition to ApoE4-associated dysfunction in AD [49], demonstrating an association between sleep quality and the presence of ApoE4 [50]. In mice, knocking out ApoE causes disturbances in circadian rhythms, with changes in the time-series expression of Bmal1 and Per, culminating in metabolic dysfunction and changes in light/dark locomotor activity [51]. Taken together, these data suggest that ApoE may be essential for the maintenance of circadian rhythms and sleep.

As both lipid metabolism and circadian rhythms are disrupted in AD and ApoE impacts both, promoting them could be a potential therapeutic strategy for AD. One potential approach to do so is time-restricted feeding/eating (TRF/TRE in humans). TRF/TRE is a behavioral intervention whereby food is only available to be consumed during the active period [52], resulting in a fasting period of at least 12 h during the inactive period and a feeding period of 12 or fewer hours during the active period. In general, TRF does not lead to caloric restriction compared to typical ad libitum feeding (ALF), where food is always available, and emphasizes when food is eaten rather than how much food is eaten. TRE can prevent and even reverse obesity and metabolic dysfunction [40,53,54,55,56,57,58,59] and pilot studies demonstrate that TRE may reduce the risk of metabolic disease among overweight or obese humans [60,61]. TRF seems to act through promoting proper metabolism, especially lipid metabolism, and circadian rhythms [54]. Recently, TRF has been proposed to prevent AD-associated dysfunction [62], and, in fact, TRF was sufficient to prevent some dysfunction in multiple amyloidosis mouse models of AD [63]. While most mechanistic TRF studies have focused on the mouse liver or *Drosophila* heart and muscle tissue [52,54,55,64], the impact of TRF on ApoE-associated dysfunction is still unknown.

Here, we use *Drosophila* to study the effects of ApoE, lipid metabolism, and TRF in the brain. The *Drosophila* system is a powerful tool for studying neurodegeneration, as expression of disease-related genes often results in disease-related phenotypes [65]. Thus, *Drosophila* is an excellent model for investigating the specificity of ApoE allele expression in the brain. Recent studies in *Drosophila* demonstrate the role of apolipoproteins in the accumulation of lipid droplets, highlighting *Drosophila* as a useful and versatile model for studying brain lipids. While *Drosophila* does not have ApoE, apolipoproteins NLaz and GLaz, which are orthologous to ApoD, can be functionally replaced by ApoE in the fly brain [11,66]. Genes related to lipid metabolism have a high homology (about 95%) with *Drosophila* genes involved in the same mechanisms, further supporting the use of this model. Our lab has previously found that TRF is beneficial in *Drosophila* models of aging, circadian disruption and diet-induced sleep, cardiac, and muscle dysfunction, and discovered novel pathways involved in these disorders that are affected by TRF [54,64,67]. Here, we determine how ApoE impacts lipid accumulation and metabolism in the brain and whether TRF could be beneficial for preventing ApoE-induced lipid dysfunction.

## 2. Materials and Methods

### 2.1. Drosophila Standard Environmental Condition and UAS-Gal4 Lines

As before [54,64,67,68], flies were housed in controlled environmental chambers set to 25 °C and 50% humidity under a 12 h light, 12 h dark cycle. Adult flies were collected at eclosion, separated by sex, female flies were held at a density of approximately 10 flies/vial and maintained on a cornmeal diet fed *ad libitum*. Flies were transferred to vials containing fresh food every 4–7 days. We used the following driver lines from the Bloomington *Drosophila* Stock Center (Bloomington, IN, USA) for cell-type specific expression: Elav-Gal4 (BL#458) for pan-neuronal expression [69] and GLaz-Gal4 (BL#23353) for glial expression [70], and Actin88f-Gal4 was courtesy of Richard Cripps for expression in flight muscles [71]. We used the following UAS lines to express different constructs: UAS-ApoE2 (BL#76604), UAS-ApoE3 (BL#76605), UAS-ApoE4 (BL#76607), UAS-DGAT2 (BL#84854), UAS-Dgat2 RNAi (Vienna *Drosophila* Research Center #107788, Vienna, Austria), and UAS-eGFP (BL#5431) for overexpression control. For driver controls, driver lines were crossed with non-transgenic w^1118^ flies. Double transgenic flies were created by first creating transgenic flies that were Elav > DGAT2 or Elav > Dgat2 RNAi with a balancer, then crossing with ApoE or control constructs.

### 2.2. Lipid-Accumulation Imaging and Analyses

Lipid accumulation was measured in a similar manner to how we have imaged lipids before [54]. Briefly, heads and thoraxes were fixed with 4% PFA in PBS for 30 min at room temperature, washed with PBS 3 × 10 min, then placed in OCT, frozen to −20 °C, and cryosectioned onto microscope slides at 20 µm for heads and 30 µm for thoraces. On each slide, heads or thoraxes from every genotype and group for a particular experimental cohort were included to account for any inter-cohort or inter-slide variability in staining intensity. Slides were washed 3 × 10 min in PBS to remove dried OCT, stained with Phalloidin-488 (1:1000 in PBS) for 30 min, washed 3 × 10 min in PBS, stained with Nile Red (1:400 in PBS) for 30 min, then washed 3 × 10 min in PBS before mounting in Prolong Diamond with DAPI. Multichannel fluorescence images were taken using Olympus BX-63 microscope at 10× using the following channels: FITC, TRITC, and DAPI. We imaged one head or thorax per image, and multiple images of different sections per fly were taken for quantification.

Lipid accumulation was quantified using ImageJ. Briefly, each multichannel image was split into three channels: FITC (green, measuring Phalloidin), TRITC (red, measuring Nile Red lipids), and DAPI (blue, measuring cell nuclei). The DAPI channel was used for region of interest selection because it allows for clear distinction between different anatomical subregions throughout the body. Regions of interest were drawn by hand in ImageJ either around the central brain, excluding the optic lobes, in the heads, or around the entire flight muscle in the thorax; representative regions of interest are shown in white in representative images. Within a region of interest, the mean fluorescent intensity of the TRITC channel was measured using ImageJ’s measure tool to quantify Nile Red staining intensity for each image. Images of multiple sections were taken for each individual fly and the mean fluorescent intensity for every section of a particular fly was averaged, resulting in an overall lipid accumulation value for one fly. After this, analysis was unblinded, and values for each fly were normalized to the average value for the youngest GFP group with constant access to food to account for variability in Nile Red staining between cohorts, then values were graphed and analyzed in Graphpad Prism version 10.3.1. (GraphPad Software, Boston, MA, USA).

### 2.3. Immunohistochemistry

Fly heads were removed using a dissecting microscope and placed in 4% PFA in PBS, spun down to ensure proper exposure to PFA for each head, and incubated for 30 min at room temperature with mild agitation. The heads were then washed 3 × 10 min in 1 × PBS and stored at 4 °C in PBS before cryosectioning. Heads were arranged in a mold with OCT and sectioned at 30 µm with a Leica CM3050 cryostat and placed on glass slides (Fisher #15-188-48). The slides were outlined with a hydrophobic pen to prepare for staining and allowed to dry for 30 min. After drying, the slides were washed 3 × 10 min with 1 × PBS to wash off dried OCT. The slides were then blocked with 3% BSA in TBST for 30 min. After this, the slides were treated with the primary antibody for APOE (sc-13521, 1:250) in 1% BSA in TBST and placed in an incubation chamber overnight at 4 °C. The next morning, the slides were washed 3 × 10 min with PBS and treated with secondary antibody (Alexaflour-594) for 1 h at room temperature. The slides were washed 3 × 10 min with 1× PBS, mounted with ProLong Diamond Antifade Mountant with DAPI (ThermoFisher (Saint Louis, MO, USA) #P36971). After drying overnight, the slides were imaged using an Olympus BX63F fluorescence microscope using CellSens Software 4.2 at 10× magnification to view one head section per image. APOE expression was analyzed using FIJI software (ImageJ v1.53v). The raw images were split into RGB channels and adjusted to minimize background fluorescence. Mean fluorescence was measured within a region of interest selected to include the brain but excluded background fluorescence in the probiscus. Average fluorescence per fly was calculated in Excel and plotted in Graphpad Prism.

### 2.4. Gene Expression by qPCR

For the qPCR the flies were flashed frozen and conserved in −80 °C until use. For RNA, the head of the flies were dissected, and the purification was made using the Zymo Quick-RNA MicroPrep Kit (#R1051, Zymo Research, Irvine, CA, USA) and all the reagents provided by the manufacturer. Briefly, we separated the heads and put in 150 µL of Lysis Buffer, conducted the lysis in room temperature, purified the minicolumns, and digested them with DNAse I for 15 min. After, the RNA was suspended in RNAse/DNAse free water and quantified using Synergy LX Multi-mode (Agilent Bio Tek Instruments, Santa Clara, CA, USA) and stored in −80 °C until use. For cDNA synthesis, we use 500 ng of RNA and 4 µL of iScript RT Supermix (#1708840, Bio-Rad Laboratories, Hercules, CA, USA). The reverse transcriptase reaction was 5 min of priming at 25 °C, 20 min of reverse transcriptase ar 46 °C, and 1 min of RT inactivation at 95 °C. To qPCR, we used 5 ng of cDNA and 200 ng of specific primers with Sso Advanced Universal SYBR Green Supermix (#1725275, Bio-Rad) in a CFX Opus Real-Time PCR System (Bio-Rad). We selected genes related to the cellular biosynthesis of cholesterol and triglycerides and searched for their orthologous genes in the *Drosophila* genome using the DRSC integrative ortholog prediction tool (DIOPT—https://www.flyrnai.org/diopt, accessed on 21 October 2024). We selected the genes that had a high similarity in the weighted score—since some genes have more than one ortholog—to design the primers. Primers for qPCR are listed below:

Dgat2-F: TGTCCAAGTTGTTGGTGCTC; Dgat2-R: GGCACTCTTCGAATTCTCCA; Srebf-F: ATGGACACGACACTGATGAAC; Srebf1-R: GACATCCTCCGCTTTGAACAA; Desat2-F: GTCGGCTACCCCTAGTCTGG; Desat2-R: TCGCCCTTGTGAATATGGAGT; Abca-F: GGAAGAACTGGACCCTCCAAT; Abca-R: AGGAGCAGGGAGAATATCGCT; Lpin-F: ACCTCGCCCATACCCAACA; Lpin-R: CCACTCCACTGATGTCCTCCA; Lsd2-F: GCTCCCTTCGTCACCAAACT; Lsd2-R: CCTGGGGTGTGTCCTTGATG; Rpl11-F: CGATCTGGGCATCAAGTACGA; Rpl11-R: TTGCGCTTCCTGTGGTTCAC; Tbp-F: ATGCCCTGAGCAACATCCAC; Tbp-R: GGATCAGCGGAACCTGGTG; Upd3-F: ATCCCCTGAAGCACCTACAGA; Upd3-R: CAGTCCAGATGCGTACTGCTG; Eiger-F: GATGGTCTGGATTCCATTGC; Eiger-R: TAGTCTGCGCCAACATCATC; Imd-F: TCAGCGACCCAAACTACAATT; Imd-R: TTGTCTGGACGTTACTGAGAGT; Dome-F: CTCACGTCTCGACTGGGAAC; Dome-R: AGAATGGTGCTTGTCAGGCA; Hop-F: CACCACCAACACCAATTC; Hop-R: GGAACGTCGTTTGGCCTTCT; Stat92e-F: CCTCGGTATGGTCACACCC; Stat92e-R: TGCCAAACTCATTGAGGGACT.

Expression was normalized with 60S ribosomal protein (Rpl11) and TATA-box binding protein (Tbp). All the results included a melting curve that was compared to a predicted in silico melting curve for the specific sequence generated by the software µMELT Quartz 3.6.2 (Available at: www.dna-utah.org/umelt/quartz/, accessed on 21 October 2024) to certify the specificity of the reaction. The results are presented as 2^−ΔΔCt^ values normalized to the expression of *Rpl11* and *Tbp* and control samples. All reactions were performed in triplicate.

### 2.5. Gene Expression Analysis of a Publicly Available Allen Institute Database

Using publicly available data from the Aging, Dementia, and TBI Study (available at: https://aging.brain-map.org/download/index, accessed on 21 October 2024), we compared gene-expression data generated by RNA sequencing from elderly individuals. The data were curated, and specific genes related to FA synthesis, elongation, saturation, cholesterol synthesis, and triglyceride synthesis were selected. RNA sequencing datasets from 107 brains were evaluated, including 377 samples from cortical parietal (PCx) and temporal (TCx) grey matter, parietal white matter (FWM), and the hippocampus (HIP). All data were organized in the form of a heatmap.

### 2.6. Time-Restricted Feeding

Flies were collected and maintained as described above in normal housing. We conducted 10 h: 14 h TRF for 2 weeks, so at 1 or 5 weeks, flies were subjected to *ad libitum* feeding (ALF) or TRF. The ALF flies were changed between vials containing food at 8 AM (lights on) and 6 PM (two hours before lights off), whereas TRF flies were in feeding vials from 8 AM to 6 PM and in vials with 1.1% agar from 6 PM to 8 AM [54,64].

### 2.7. Statistical Analysis

We used ordinary least-squares (OLS) regression to assess deviations in gene expression across four brain regions (HIP, PCx, TCx, and FWM) from the global mean expression (average expression across all regions). The model included brain region as a categorical predictor, and coefficients (β) with 95% confidence intervals (CIs) were estimated. Statistical significance was set at *p* < 0.05. All analyses were performed in Python using the statsmodels package. Significance in brain lipid accumulation was determined using one- or two-way ANOVA with post hoc Sidak’s method for multiple comparisons. For qPCR analysis, differences between samples were determined using two-way or three-way ANOVA with post hoc Fisher LSD method. Bar graphs show mean ± SEM.

## 3. Results

### 3.1. ApoE Induces Lipid Accumulation in the Brain but Not in the Indirect Flight Muscle

**Figure 1:**
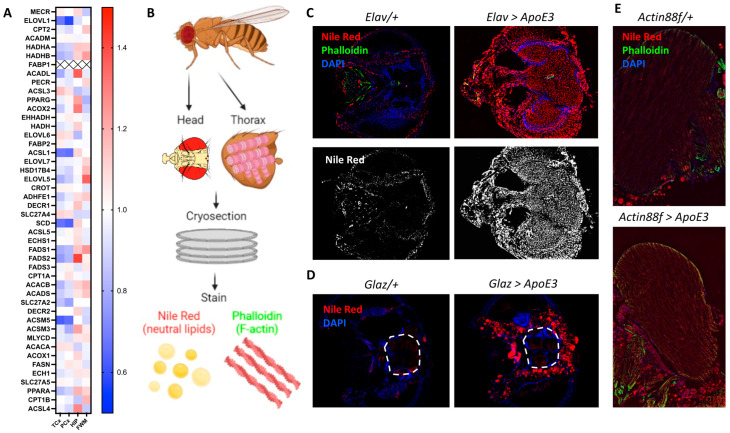
ApoE3 induces lipid accumulation in the head. (**A**): Gene expression related to cellular biosynthesis selected using the KEGG pathway #hsa01212, showing genes regulators of saturation, and elongation of fatty acids, as well as triglyceride and cholesterol synthesis (Fold), in the hippocampus (HIP) as well as in the parietal cortex (PCx), temporal cortex (TCx), and parietal white matter (FWM). (**B**): Schematic of experimental design. Adult Drosophila heads and thoraxes were isolated and fixed with paraformaldehyde. The tissue was then cryosectioned and stained with Nile Red to view neutral lipids and phalloidin to view F-actin. (**C**): Representative images demonstrate that when driven in all neurons with the Elav-Gal4 driver, ApoE3 induces lipid accumulation throughout the head, including the brain, compared to driver control (*Elav*/+). (**D**): Representative images demonstrate that when driven in glia with the GLaz-Gal4 driver, ApoE3 induces lipid accumulation in the head, but not the neurons of the brain compared to driver control (*GLaz*/+). (**E**): Representative images demonstrate that when driven in flight muscles with the Actin88f-Gal4 driver, ApoE3 does not induce lipid accumulation compared to driver control (*Actin88f*/+).

Extensive literature demonstrates that ApoE is the largest genetic risk factor for Alzheimer’s disease (AD). However, our understanding of the functions of ApoE and the potential mechanisms through which different alleles can influence AD outcomes remains limited. To address this knowledge gap, we conducted an in-depth analysis of the Allen Institute’s database examining expression patterns of lipid synthesis-related proteins in different regions of the brain among elderly patients (Figure 1A). We found significant variations in protein expression, with increased levels of many genes in the hippocampus, supporting the importance of lipids and lipid synthesis in memory [3,4]. To confirm the qualitative visualization, we used an ordinary least-squares (OLS) regression model to assess how gene-expression patterns in different brain regions deviate from the global mean expression. The hippocampus (HIP) exhibited a significant positive deviation (β = 0.051, *p* < 0.001), while the parietal cortex (PCx) and temporal cortex (TCx) exhibited significant negative deviations (PCx: β = −0.124, *p* < 0.001; TCx: β = −0.136, *p* < 0.001). These results suggest that the gene expression of lipid metabolism-related genes in the hippocampus is higher compared to the average expression across all brain regions.

To mechanistically study the impact of ApoE in the brain, we utilized the *UAS-Gal4* system [72] to express ApoE3, the common allele in humans, in different cell types in *Drosophila*. After expressing ApoE3 in a particular tissue, we collected the head and thorax, cryosectioned the tissue, and stained for neutral lipids with Nile Red and for actin filaments with phalloidin (Figure 1B). First, we expressed ApoE3 pan-neuronally with the *Elav-Gal4* driver and used immunohistochemistry to confirm the expression of ApoE3 in the brain (Supplementary Figure S1). We found that ApoE3 drove lipid accumulation throughout the head and brain (Figure 1C). As ApoE is highly expressed in the glia in the human brain under normal conditions, we next expressed ApoE3 in some glia using the *Glaz-Gal4* driver. Interestingly, we found that while glial expression of ApoE3 induced lipid accumulation around the brain, where there are significant glial populations in *Drosophila* [73,74], it did not cause significant lipid accumulation in the neuropil (Figure 1D). As it was unsurprising that overexpressing a lipoprotein in a highly metabolic tissue led to lipid deposition in areas where those cells predominate, we next determined if this is always the case. We expressed ApoE3, the most common allele, in the flight muscle, which is highly metabolically active like the brain, using the *Actin88f-Gal4* driver. To our surprise, there was not significantly more lipid accumulation in flight muscles in the thorax (Figure 1E), suggesting that ApoE3’s effect on lipid accumulation may be specific to the head. Additionally, we tested whether pan-neuronal ApoE had any non-cell-autonomous lipid accumulation and found that neuronal ApoE3 did not cause lipid accumulation in flight muscles (Supplementary Figure S2). Altogether, we found that expressing ApoE3 in the head led to lipid accumulation in the head but not in peripheral tissue.

A: Gene expression related to cellular biosynthesis selected using the KEGG pathway #hsa01212, showing gene regulators of saturation and elongation of fatty acids, as well as triglyceride and cholesterol synthesis (Fold), in the hippocampus (HIP), as well as in the parietal cortex (PCx), temporal cortex (TCx), and parietal white matter (FWM). B: Schematic of experimental design. Adult *Drosophila* heads and thoraxes were isolated and fixed with paraformaldehyde. The tissue was then cryosectioned and stained with Nile Red to view neutral lipids and phalloidin to view F-actin. C: Representative images demonstrate that when driven in all neurons with the *Elav-Gal4* driver, ApoE3 induces lipid accumulation throughout the head, including the brain, compared to driver control (*Elav*/+). D: Representative images demonstrate that when driven in the glia with the *GLaz-Gal4* driver, ApoE3 induces lipid accumulation in the head, but not the neurons of the brain compared to driver control (*GLaz*/+). E: Representative images demonstrate that when driven in flight muscles with the *Actin88f-Gal4* driver, ApoE3 does not induce lipid accumulation compared to driver control (*Actin88f*/+).

### 3.2. All ApoE Alleles Induce Similar Lipid Accumulation in the Brain

**Figure 2:**
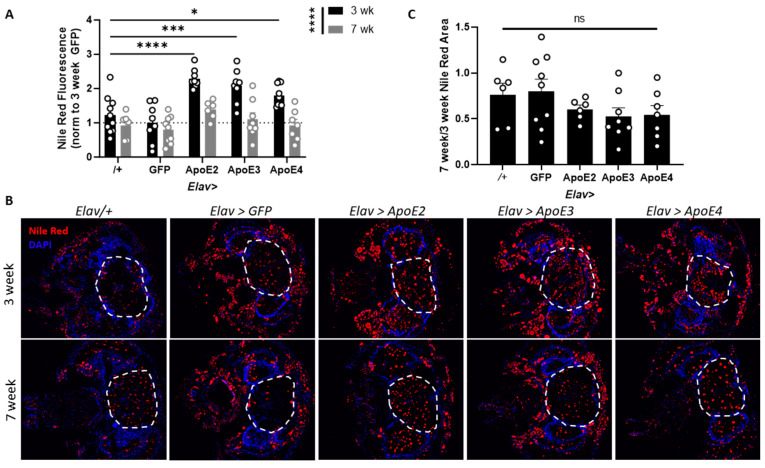
All ApoE alleles induce lipid accumulation in the brain. (**A**) Quantification of lipid accumulation in the brains of flies at 3 and 7 weeks of age demonstrates that ApoE induces lipid accumulation in the brain, and old flies have less lipid accumulation than young flies (2-way ANOVA main effect of aging F(1,70) = 42.7, *p* < 0.0001, Sidak posthoc * *p* < 0.05, *** *p* < 0.001, **** *p* < 0.0001 compared to 3-week-old driver control (*Elav*/+), n 6–10 flies per group). (**B**) Representative images of flies showing an increase in lipids in the brains of *Elav > ApoE2/3/4* flies at a young age and a decrease in lipid accumulation with age. (**C**) The ratio of lipid accumulation of old to young flies shows no ApoE- or allele-dependent differences in lipid loss with age (ANOVA F (4,31) = 1.4, *p* = 0.266, ns = not significant, n = 6–9 flies per group). Dashed lines represent regions of interest for quantification.

Next, we determined whether there were alleles- and age-dependent effects of neuronal ApoE in the brain. We expressed each ApoE allele in neurons and measured lipid accumulation at 3 weeks of age (early middle age) and 7 weeks of age (older age). We found that each allele of ApoE significantly increased lipid accumulation in the brain compared to control flies at 3 weeks of age and that lipids decreased in the brain with age (Figure 2A,B). However, this age effect is likely due to less expression of ApoE in aged flies (Supplementary Figure S1). To determine whether there are any ApoE or its alleles-modified age-induced lipid decreases, we measured the ratio of lipid accumulation at 7 weeks to lipid accumulation at 3 weeks for each genotype. We found that there was no significant difference in the effect of aging between genotypes (Figure 2C). Thus, our results show that each ApoE allele causes lipid accumulation in *Drosophila* brains and that lipid accumulation decreases with age regardless of genotype, possibly due to less expression of ApoE with age.

### 3.3. Pan-Neuronal Knock-Down of Dgat2 Prevents Lipid Accumulation in ApoE Allele-Specific Manner

**Figure 3:**
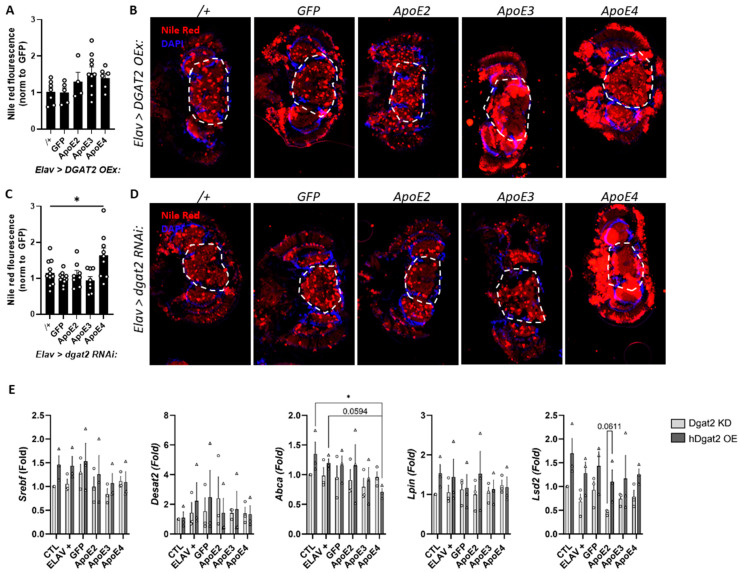
Dgat2 is required for ApoE2- and ApoE3-induced lipid accumulation. (**A**) Quantification of lipid accumulation in the brains of flies overexpressing DGAT2 shows that ApoE still induces lipid accumulation (n = 4–10 flies per group). (**B**) Representative images of heads of flies overexpressing DGAT2 and ApoE. (**C**) Quantification of lipid accumulation in the brains of flies with Dgat2 RNAi shows that lipid accumulation only increases with ApoE4 overexpression, but not ApoE2/3 (ANOVA F(4,42) = 4.6, *p* = 0.004, Sidak post-hoc * *p* < 0.05 relative to Dgat2 RNAi (*Elav > Dgat2 RNAi*/+), n = 8–11 flies per group). (**D**) Representative images of heads of flies with *Dgat2* RNAi and ApoE overexpression show the increase in lipid accumulation only in *Elav > Dgat2 RNAi: ApoE4* flies. (**E**) Effects of Dgat2 modulation on expression of genes related to fatty acid and triglyceride biosynthesis in the head. The data were normalized compared to a control group (Elav/+) and compared to both Elav/+ and Elav > GFP controls (CTL). (2-way ANOVA main effect of Dgat2 on Lsd2 (1, 28) = 18.23, and on Srebf (1, 24) = 4.276. Fisher LSD post-hoc * *p* < 0.05. Dashed lines represent regions of interest for quantification.

One gene that is critical for lipid metabolism throughout the body is diacylglycerol O-acyltransferase 2 (*Dgat2*), an enzyme that catalyzes the final step in triglyceride synthesis, which can be stained with Nile Red. Dgat2 has also been associated with ApoE and lipid droplet deposition [75], and could provide a link between ApoE, lipid metabolism, and lipid accumulation. Thus, we wanted to determine whether Dgat2 modifies ApoE-induced lipid accumulation. First, we determined whether manipulating neuronal *Dgat2* levels affected lipid accumulation in the brain. We knocked down *Drosophila dgat2* or overexpressed human *DGAT2* and compared it to appropriate controls, namely an empty RNAi line and GFP overexpression, and found that there were no significant differences in lipid accumulation (Supplementary Figure S3). We next created double transgenic flies that both express different ApoE alleles and either overexpress human *DGAT2* or contain RNAi to knock down *Drosophila Dgat2*. We did not find a modification of ApoE-induced lipid accumulation when DGAT2 is overexpressed (Figure 3A,B). In contrast, we found that knocking down *Dgat2* prevented lipid accumulation in flies overexpressing ApoE2 and ApoE3, but not ApoE4 (Figure 3C,D). We did not see any differences in lipid accumulation between control flies with *DGAT2* overexpression and *Dgat2* RNAi, suggesting that modulating Dgat2 levels alone did not have a significant effect on lipid accumulation in the brain. Together, these results suggest that Dgat2 is required for ApoE-induced lipid accumulation in an allele-specific manner, where ApoE4 is able to induce lipid accumulation even without Dgat2.

### 3.4. Impact of Dgat2 on Intracellular Biosynthesis of Lipids with the Expression of ApoE Allele

To further explore the effects of *Dgat2* on ApoE, we analyzed the expression of key genes involved in the intracellular biosynthesis of lipids. We found that modulating *Dgat2* expression significantly changed the brain expression of *Srebf*, the main regulator of lipid homeostasis, and *Lsd2*, a protein present in lipid droplets, which is consistent with the involvement of *Dgat2* in lipid droplet formation (Figure 3E). However, we did not observe allele-dependent changes in the expression of these genes, suggesting that the role of *ApoE4* in lipid accumulation may occur at the post-transcriptional level or through clearance of FAs from the cell and be independent of *Dgat2* gene expression. There were no differences in the expression of *Lpin* and *Desat2*, indicating no changes in FA structure, and no difference in *Abca* expression, indicating no influence of *Dgat2* in the *Abca*-related lipidation of *ApoE*.

### 3.5. Time-Restricted Feeding Prevents ApoE-Induced Lipid Accumulation in the Brain

**Figure 4:**
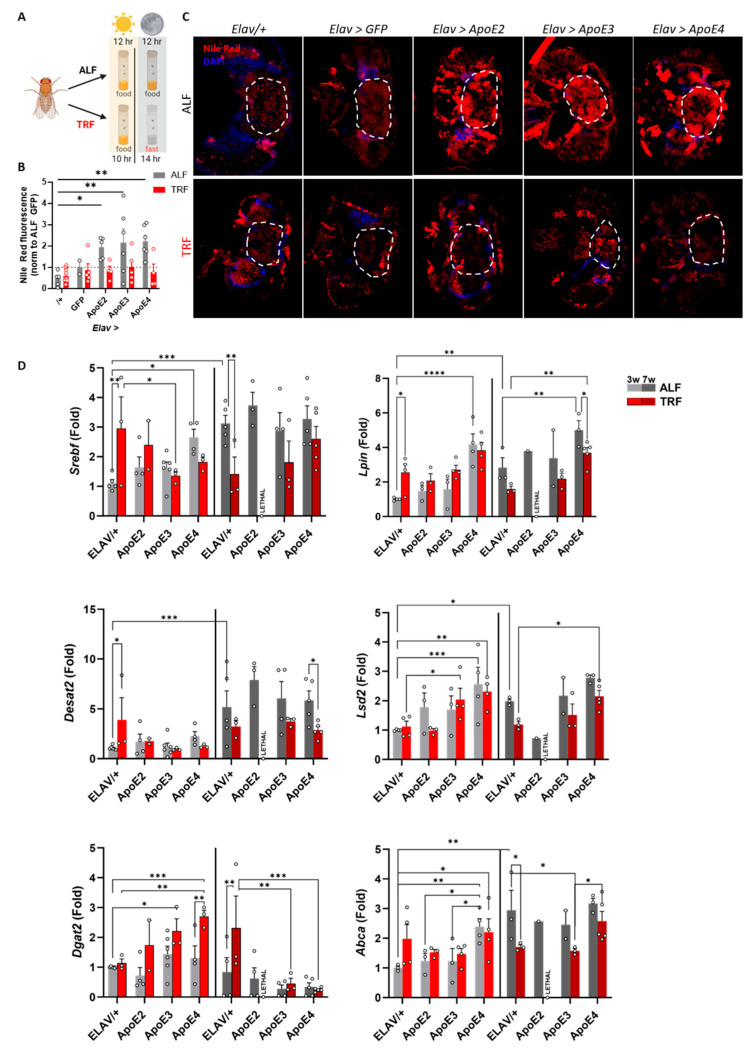
Time-restricted feeding prevents ApoE-induced lipid accumulation in the brain. (**A**) Schematic of TRF paradigm. Flies were split into ALF and TRF groups, all were kept on 12 h:12 h light:dark cycles. ALF flies always had access to food, while TRF flies only had access to food during the first 10 h of the light period and fasted for 14 h. (**B**) Quantification of lipid accumulation in the brain shows that TRF prevents ApoE-induced lipid accumulation in the brains of flies (2-way ANOVA main effect of TRF F(1,44) = 11.3, Sidak post-hoc * *p* < 0.05, ** *p* < 0.01 relative ALF driver control (*Elav*/+), n = 2–12 per group). (**C**) Representative images of heads of flies show that under ALF, ApoE2/3/4 induces lipid accumulation, while under TRF, ApoE does not induce lipid accumulation. (**D**) Effects of time-restricted feeding (TRF) on the expression of genes associated with fatty acid and triglyceride biosynthesis in adult (3 weeks old) and old-adult (7 weeks old) flies head. Three-way Anova and mixed-effects analysis was conducted, as well as post-hoc Fisher LSD test (* *p* < 0.05; ** *p* < 0.01; *** *p* < 0.001; **** *p* < 0.0001). Dashed lines represent regions of interest for quantification.

Currently, there are no approved therapeutic strategies that target ApoE-associated dysfunction in AD, which is the leading genetic risk factor for late-onset AD. One attractive therapeutic approach to ameliorate metabolic dysfunction is time-restricted feeding (TRF), which we have previously found is beneficial for metabolic disorders and muscle function [54,64,67]. We also found that TRF-mediated inhibition of *Dgat*2 prevents lipid metabolism dysfunction in the periphery and prevents age and obesity-associated dysfunction [67]. Thus, we wanted to determine if TRF could prevent ApoE-induced dysfunction. We split flies into two groups, *ad libitum* fed (ALF) flies and TRF flies, both kept on 12 h light–dark cycles. Starting at one week of age, ALF flies were allowed constant access to food, while TRF flies only had access to food for the first 10 h of the light period but fasted for 14 h (Figure 4A). We found that two weeks of TRF prevented ApoE-induced lipid accumulation in all genotypes without affecting lipid accumulation in the control groups (Figure 4B,C). To our knowledge, this is the first evidence that TRF prevents ApoE-associated dysfunction, supporting the idea that it could be beneficial in the context of AD.

### 3.6. Time-Restricted Feeding Modulates ApoE-Induced Genes Linked with Lipid Dysmetabolism

We next wanted to determine the effects of TRF on the expression of lipoproteins within the brain. To do this, we utilized our previously published dataset of time course RNAseq from nontransgenic flies at 3 weeks of age after completing 2 weeks of TRF [64], which is the same paradigm we use here. Specifically, we examined the expression of apolipoproteins *Nlaz* and *Glaz*, which are human *ApoD* orthologs, share sequence similarity with human *ApoE*, and can be functionally replaced by human ApoE in *Drosophila* [11]. We found that TRF shifted the peak expression of both genes by 12 h: *Glaz* from ZT18 to ZT6 and *Nlaz* from ZT0 to ZT12 (Supplementary Figure S4), demonstrating a clear effect of TRF on rhythmic transcription of endogenous apolipoproteins.

We also performed qPCR to determine the transcriptional effects of TRF on genes associated with lipid metabolism in our flies (Figure 4D). First, we found a statistically significant aging effect on the expression levels of *Dgat2* (F(1, 44) = 22.24, *p* < 0.0001), *Desat2* (F(1, 44) = 22.61, *p* < 0.0001), and *Abca* (F(1, 33) = 6.028, *p* = 0.05). Second, TRF significantly impacted the expression of *Dgat2*, *Srebf*, *Desat2*, and *Abca*. Third, a genotype effect was observed on the expression of *Abca* (F(3, 33) = 7.539, *p* < 0.001), *Lpin* (F(3, 34) = 19.65, *p* < 0.0001), and *Lsd2* (F(3, 34) = 13.79, *p* < 0.0001). Fourth, interactions were detected between genotype and age on *Dgat2* expression (F(3, 44) = 8.246, *p* < 0.001). Finally, an interaction between age and TRF was observed on the expression levels of *Srebf*, (F (1, 43) = 19.44, *p* < 0.0001), *Desat2* (F(1, 44) = 14.72, *p* < 0.001), *Abca* (F(1, 33) = 16.83, *p* < 0.001), and *Lpin* (F(1, 34) = 22.67, *p* < 0.0001). The interaction between age, TRF, and genotype was found in Srebf (F(3, 43) = 5.018, *p* < 0.01) and Dgat2 (F(3, 44) = 3.533, *p* < 0.05). Additional statistical data are shown in Supplementary Document S2. These findings highlight the complex interplay between aging, genotype, and TRF in modulating the expression of key genes involved in lipid metabolism. Specifically, we observed an age-dependent effect on genes involved in lipid biosynthesis, with increased expression of the majority of these genes associated with aging. At the same time, TRF resulted in decreased expression of these genes. This supports the idea that TRF can prevent aging-associated dysfunction in lipid metabolism. In 3-week-old flies, we observed an allele-dependent shift, where the expression of ApoE4 specifically increased the expression of *Srebf*, *Lpin*, *Lsd2*, and *Abca*. In aged flies, TRF reduced the expression of *Desat2* and *Lpin* in ApoE4 flies, while no changes were observed in *Dgat2* expression, suggesting that TRF-induced modulation of lipid accumulation may act upstream of *Dgat2*. Interestingly, *Dgat2* exhibited an opposite pattern to the other genes, decreasing with aging and increasing with TRF, indicating its regulatory role in lipid metabolism and accumulation. Additionally, as inflammation is key in AD progression [76], we also analyzed the expression of selected neuroinflammatory markers but did not see a clear effect of the different ApoE alleles and TRF on inflammation (Supplementary Figure S5). Altogether, our results demonstrate that TRF prevents ApoE-induced lipid accumulation, likely by modifying lipid metabolism genes.

## 4. Discussion

Here, we report that lipid metabolism genes are differentially expressed in different regions of the human brain, with an increased expression in the hippocampus, which is selectively vulnerable to neurodegeneration in AD. Next, we studied the effect of ApoE on lipids in the brain and found that ApoE induces lipid accumulation that decreases with age specifically in the brain. We note that there was a difference in expression between the alleles, which is a common caveat to most overexpression systems in animal models, including the UAS–Gal4 system in *Drosophila* [77]. While ApoE2 and ApoE4 had similar overexpression levels, ApoE3 had relatively lower overexpression levels (Supplementary Figure S1), which could affect the interpretation of the results. We do note that overexpressing all three alleles induced significant lipid accumulation in the brain that did not significantly differ between alleles (Figure 2), suggesting that only a little ApoE is needed to induce lipid accumulation that may have a rapid ceiling effect for all three alleles. Knocking down expression of *Dgat2* prevented ApoE2- and ApoE3-induced lipid accumulation. Recent studies show that in the liver, the inhibition of Dgat2 blocks SREBP-1 cleavage and decreases *Srebf1* gene expression, reducing lipogenesis [78,79]. Our results support the idea that in the fly neuron, knocking down Dgat2 has the same effect and inhibits *Srebf*, the only ortholog of human SREBF1 and SREBF2, and a master regulator of FA biogenesis. However, ApoE4 was still able to induce lipid accumulation independent of *Dgat2*. ApoE is responsible for coupling the FA metabolism of astrocytes and neurons. While it transfers cholesterol to neurons, it is responsible for transferring FA from neurons to astrocytes. ApoE4 has a decreased capacity for FA transferring, which can explain the LD accumulation even with reduced Dgat2 [13]. Finally, we found that TRF prevents ApoE-induced lipid accumulation, likely through the regulation of lipid metabolism genes like *Lsd2*, *Desat2*, *Srebf*, and *Lpin*. Our findings demonstrate that ApoE plays a significant role in lipid metabolism within neurons, and different ApoE alleles can lead to alterations in the expression of genes involved in the regulation of intracellular FA and cholesterol synthesis, as well as lipid accumulation. Importantly, TRF was sufficient to prevent ApoE-associated dysfunction, highlighting it as a potential therapeutic strategy for preventing lipid dysfunction associated with aging and AD.

We observed an age-related effect on the upregulation of gene expression associated with lipid biosynthesis. Some of these genes were already increased in ApoE4 flies, which could be indicative of premature aging in these flies. Importantly, the upregulation of certain genes is linked to AD. Recent studies have demonstrated that the use of *Scd-1*, a crucial regulator of FA desaturation (the human ortholog of *Drosophila Desat2*), inhibitors improves memory in 3× Tg AD mice independent of amyloid plaque formation and neurofibrillary tangles [80]. In flies, we demonstrated that aging independently increases the expression of *Desat2*, while TRF led to a decrease in its expression. *Desat2*/*SCD-1* is the rate-limiting enzyme in the conversion of saturated FA to delta-9 monounsaturated fatty acids (MUFA), and both SCD-1 and MUFA are increased in the brains of AD patients [81].

We also found an increased expression of *Lpin* in ApoE4 flies, an enzyme that catalyzes the formation of diacylglycerol from phosphatidate within the cell. Importantly, aberrant accumulation of diacylglycerol occurs in AD, and it has been found in both serum and post-mortem analyses of the frontal cortex in patients [82]. Similarly to *Desat2*, our results also show that ApoE4 leads to an increased expression of *Lpin*, which is also reversed by TRF. In addition to diacylglycerol serving as a substrate for *Dgat2*, several factors indicate a preference for *Dgat2* for diacylglycerols rich in MUFA, which are products of *Desat2*/*Scd1*. Notably, both proteins co-localize in the endoplasmic reticulum, and these types of FA are predominant in triacylglycerols. Inhibition of Scd1 in mice also results in a decrease in triglycerides [83]. Thus, our results support the idea that lipid metabolism is important in aging and AD, and that TRF could be beneficial to prevent these associated dysfunctions.

Safe and efficacious therapeutic strategies for AD are desperately needed as an aging worldwide population will result in dramatic growth of the disease [1]. Recent successes in phase III clinical trials showing that amyloid-β targeting antibodies lecanemab [84] and donanemab (unpublished statement from Eli Lilly, Indianapolis, IN, USA) can slow cognitive decline in AD are promising. However, safety concerns, especially for ApoE4 carriers [85], and cost [86] could be significant barriers to treatment for many people. Thus, our findings that TRF can prevent ApoE-associated dysfunction in the brain opens a new avenue for therapeutic development. To our knowledge, this is the first interventional study showing that TRF can prevent ApoE-associated dysfunction in preclinical models and is supported by a preliminary poster claiming that TRF can prevent sleep disruption in a mouse model of AD [87]. TRF prevented lipid accumulation even in ApoE4 flies, suggesting that its beneficial effects go beyond that of targeting *Dgat2*, and that TRF may be more beneficial than therapeutic strategies that only target one part of lipid metabolism rather than targeting lipid metabolism along with circadian dysfunction. TRF increases the activity of AMPK [67], while TRF-mediated AMPK activation can lead to the inhibition of FA production by inhibition of Acetyl-COA carboxylase (ACC) [88]. ACC is upstream of *Dgat2* and could explain the diminished LD production, even in ApoE4 flies. Our findings complement the recent report that TRF can reduce pathology and slow Aβ-induced cognitive decline in mice [63], suggesting that TRF may benefit multiple aspects of AD-associated dysfunction. Importantly, TRF would be significantly more affordable than current or projected AD therapeutics, as it only requires changing when one eats and, thus, has tremendous therapeutic potential if it prevents dysfunction in AD. This would prevent the barrier of cost for many poorer individuals who bear a disproportionate burden in AD, and for those in the developing countries that are projected to have the highest growth rate of AD [1].

In total, our data demonstrate that TRF decreases ApoE-related lipid accumulation by modulating the expression of *Lpin* and *Desat2*, thereby influencing the activity of Dgat2 and reducing lipid accumulation. We support the importance of lipid metabolism in the brain during aging and AD and tie ApoE directly to lipid accumulation. We add to the body of evidence that lipid metabolism is critical for brain function in the context of AD and highlight TRF as a new potential therapeutic avenue for preventing dysfunction in AD models.

## Data Availability

All datasets produced for this publication are included in the figures and raw data is available upon reasonable request.

## Supplementary Materials

The following supporting information can be downloaded at: https://www.mdpi.com/article/10.3390/genes15111376/s1, Supplementary Figures S1–S5 are attached in a separate document (Supplementary Document S1). Supplementary Document S2: Statistical report of Figure 4D, with comprehensive statistics of the three-way ANOVA and posthoc test performed to evaluate the effects and interactions between TRF, age and genotype on gene expression levels (Dgat2, Srebf, Desat2, Abca, Lpin and Lsd2).

## References

[1] Collaborators G.D.F. (2022). Estimation of the global prevalence of dementia in 2019 and forecasted prevalence in 2050: An analysis for the Global Burden of Disease Study 2019. Lancet Public Health.

[2] Corder E.H., Saunders A.M., Strittmatter W.J., Schmechel D.E., Gaskell P.C., Small G.W., Roses A.D., Haines J.L., Pericak-Vance M.A. (1993). Gene dose of apolipoprotein E type 4 allele and the risk of Alzheimer’s disease in late onset families. Science.

[3] Oitzl M.S., Mulder M., Lucassen P.J., Havekes L.M., Grootendorst J., de Kloet E.R. (1997). Severe learning deficits in apolipoprotein E-knockout mice in a water maze task. Brain Res..

[4] Grootendorst J., Bour A., Vogel E., Kelche C., Sullivan P.M., Dodart J.C., Bales K., Mathis C. (2005). Human apoE targeted replacement mouse lines: H-apoE4 and h-apoE3 mice differ on spatial memory performance and avoidance behavior. Behav. Brain Res..

[5] Lanfranco M.F., Sepulveda J., Kopetsky G., Rebeck G.W. (2021). Expression and secretion of apoE isoforms in astrocytes and microglia during inflammation. Glia.

[6] Metzger R.E., LaDu M.J., Pan J.B., Getz G.S., Frail D.E., Falduto M.T. (1996). Neurons of the human frontal cortex display apolipoprotein E immunoreactivity: Implications for Alzheimer’s disease. J. Neuropathol. Exp. Neurol..

[7] Boschert U., Merlo-Pich E., Higgins G., Roses A., Catsicas S. (1999). Apolipoprotein E expression by neurons surviving excitotoxic stress. Neurobiol. Dis..

[8] Koutsodendris N., Blumenfeld J., Agrawal A., Traglia M., Grone B., Zilberter M., Yip O., Rao A., Nelson M.R., Hao Y. (2023). Neuronal APOE4 removal protects against tau-mediated gliosis, neurodegeneration and myelin deficits. Nat. Aging.

[9] Korinek M., Gonzalez-Gonzalez I.M., Smejkalova T., Hajdukovic D., Skrenkova K., Krusek J., Horak M., Vyklicky L. (2020). Cholesterol modulates presynaptic and postsynaptic properties of excitatory synaptic transmission. Sci. Rep..

[10] Koudinov A.R., Koudinova N.V. (2001). Essential role for cholesterol in synaptic plasticity and neuronal degeneration. FASEB J..

[11] Liu L., MacKenzie K.R., Putluri N., Maletić-Savatić M., Bellen H.J. (2017). The Glia-Neuron Lactate Shuttle and Elevated ROS Promote Lipid Synthesis in Neurons and Lipid Droplet Accumulation in Glia via APOE/D. Cell Metab..

[12] Ioannou M.S., Jackson J., Sheu S.-H., Chang C.-L., Weigel A.V., Liu H., Pasolli H.A., Xu C.S., Pang S., Matthies D. (2019). Neuron-astrocyte metabolic coupling protects against activity-induced fatty acid toxicity. Cell.

[13] Qi G., Mi Y., Shi X., Gu H., Brinton R.D., Yin F. (2021). ApoE4 Impairs Neuron-Astrocyte Coupling of Fatty Acid Metabolism. Cell Rep..

[14] Farmer B.C., Kluemper J., Johnson L.A. (2019). Apolipoprotein E4 Alters Astrocyte Fatty Acid Metabolism and Lipid Droplet Formation. Cells.

[15] Alzheimer A. (1907). Uber eigenartige Erkrankung der Hirnrinde. Allg. Z. Für Psychiatr. Und Psych. Gerichtl. Med..

[16] Fuller S.C. (1912). Alzheimer’s disease (senium praecox): The report of a case and review of published cases. J. Nerv. Ment. Dis..

[17] Wightman D.P., Jansen I.E., Savage J.E., Shadrin A.A., Bahrami S., Holland D., Rongve A., Børte S., Winsvold B.S., Drange O.K. (2021). A genome-wide association study with 1,126,563 individuals identifies new risk loci for Alzheimer’s disease. Nat. Genet..

[18] Bellenguez C., Küçükali F., Jansen I.E., Kleineidam L., Moreno-Grau S., Amin N., Naj A.C., Campos-Martin R., Grenier-Boley B., Andrade V. (2022). New insights into the genetic etiology of Alzheimer’s disease and related dementias. Nat. Genet..

[19] Depp C., Sun T., Sasmita A.O., Spieth L., Berghoff S.A., Nazarenko T., Overhoff K., Steixner-Kumar A.A., Subramanian S., Arinrad S. (2023). Myelin dysfunction drives amyloid-β deposition in models of Alzheimer’s disease. Nature.

[20] Kao Y.H., Chou M.C., Chen C.H., Yang Y.H. (2019). White Matter Changes in Patients with Alzheimer’s Disease and Associated Factors. J. Clin. Med..

[21] Area-Gomez E., Larrea D., Pera M., Agrawal R.R., Guilfoyle D.N., Pirhaji L., Shannon K., Arain H.A., Ashok A., Chen Q. (2020). APOE4 is associated with differential regional vulnerability to bioenergetic deficits in aged APOE mice. Sci. Rep..

[22] Zhao N., Ren Y., Yamazaki Y., Qiao W., Li F., Felton L.M., Mahmoudiandehkordi S., Kueider-Paisley A., Sonoustoun B., Arnold M. (2020). Alzheimer’s risk factors age, APOE genotype, and sex drive distinct molecular pathways. Neuron.

[23] Miranda A.M., Ashok A., Chan R.B., Zhou B., Xu Y., McIntire L.B., Area-Gomez E., Di Paolo G., Duff K.E., Oliveira T.G. (2022). Effects of APOE4 allelic dosage on lipidomic signatures in the entorhinal cortex of aged mice. Transl. Psychiatry.

[24] Roher A.E., Maarouf C.L., Sue L.I., Hu Y., Wilson J., Beach T.G. (2009). Proteomics-derived cerebrospinal fluid markers of autopsy-confirmed Alzheimer’s disease. Biomarkers.

[25] Yang L.G., March Z.M., Stephenson R.A., Narayan P.S. (2023). Apolipoprotein E in lipid metabolism and neurodegenerative disease. Trends Endocrinol. Metab..

[26] Tao Q., Ang T.F.A., DeCarli C., Auerbach S.H., Devine S., Stein T.D., Zhang X., Massaro J., Au R., Qiu W.Q. (2018). Association of Chronic Low-grade Inflammation with Risk of Alzheimer Disease in ApoE4 Carriers. JAMA Netw. Open.

[27] Arnaud L., Benech P., Greetham L., Stephan D., Jimenez A., Jullien N., García-González L., Tsvetkov P.O., Devred F., Sancho-Martinez I. (2022). APOE4 drives inflammation in human astrocytes via TAGLN3 repression and NF-κB activation. Cell Rep..

[28] Harmer S.L., Panda S., Kay S.A. (2001). Molecular bases of circadian rhythms. Annu. Rev. Cell Dev. Biol..

[29] Panda S., Hogenesch J.B., Kay S.A. (2002). Circadian rhythms from flies to human. Nature.

[30] Reppert S.M., Weaver D.R. (2000). Comparing clockworks: Mouse versus fly. J. Biol. Rhythms..

[31] Hardin P.E., Panda S. (2013). Circadian timekeeping and output mechanisms in animals. Curr. Opin. Neurobiol..

[32] Duffy J.F., Czeisler C.A. (2002). Age-related change in the relationship between circadian period, circadian phase, and diurnal preference in humans. Neurosci Lett..

[33] Watanabe A., Shibata S., Watanabe S. (1995). Circadian rhythm of spontaneous neuronal activity in the suprachiasmatic nucleus of old hamster in vitro. Brain Res..

[34] Satinoff E., Li H., Tcheng T.K., Liu C., McArthur A.J., Medanic M., Gillette M.U. (1993). Do the suprachiasmatic nuclei oscillate in old rats as they do in young ones?. Am. J. Physiol..

[35] Aujard F., Cayetanot F., Bentivoglio M., Perret M. (2006). Age-related effects on the biological clock and its behavioral output in a primate. Chronobiol. Int..

[36] Aujard F., Herzog E.D., Block G.D. (2001). Circadian rhythms in firing rate of individual suprachiasmatic nucleus neurons from adult and middle-aged mice. Neuroscience.

[37] Musiek E.S., Bhimasani M., Zangrilli M.A., Morris J.C., Holtzman D.M., Ju Y.S. (2018). Circadian Rest-Activity Pattern Changes in Aging and Preclinical Alzheimer Disease. JAMA Neurol..

[38] Milan-Tomas A., Shapiro C.M. (2018). Circadian Rhythms Disturbances in Alzheimer Disease: Current Concepts, Diagnosis, and Management. Alzheimer’s Dis. Assoc. Disord..

[39] Stevanovic K., Yunus A., Joly-Amado A., Gordon M., Morgan D., Gulick D., Gamsby J. (2017). Disruption of normal circadian clock function in a mouse model of tauopathy. Exp. Neurol..

[40] Manoogian E.N.C., Panda S. (2017). Circadian rhythms, time-restricted feeding, and healthy aging. Ageing Res. Rev..

[41] Harper D.G., Stopa E.G., McKee A.C., Satlin A., Fish D., Volicer L. (2004). Dementia severity and Lewy bodies affect circadian rhythms in Alzheimer disease. Neurobiol. Aging.

[42] Tate B., Aboody-Guterman K.S., Morris A.M., Walcott E.C., Majocha R.E., Marotta C.A. (1992). Disruption of circadian regulation by brain grafts that overexpress Alzheimer beta/A4 amyloid. Proc. Natl. Acad. Sci. USA.

[43] Touitou Y., Reinberg A., Bogdan A., Auzeby A., Beck H., Touitou C. (1986). Age-related changes in both circadian and seasonal rhythms of rectal temperature with special reference to senile dementia of Alzheimer type. Gerontology.

[44] van Himbergen T.M., Beiser A.S., Ai M., Seshadri S., Otokozawa S., Au R., Thongtang N., Wolf P.A., Schaefer E.J. (2012). Biomarkers for insulin resistance and inflammation and the risk for all-cause dementia and alzheimer disease: Results from the Framingham Heart Study. Arch. Neurol..

[45] Thome J., Coogan A.N., Woods A.G., Darie C.C., Hassler F. (2011). CLOCK Genes and Circadian Rhythmicity in Alzheimer Disease. J. Aging Res..

[46] Cermakian N., Lamont E.W., Boudreau P., Boivin D.B. (2011). Circadian clock gene expression in brain regions of Alzheimer’s disease patients and control subjects. J. Biol. Rhythms..

[47] Gottlieb D.J., DeStefano A.L., Foley D.J., Mignot E., Redline S., Givelber R.J., Young T. (2004). *APOE ε4* is associated with obstructive sleep apnea/hypopnea. Sleep Heart Health Study.

[48] Basta M., Zaganas I., Simos P., Koutentaki E., Dimovasili C., Mathioudakis L., Bourbouli M., Panagiotakis S., Kapetanaki S., Vgontzas A. (2021). Apolipoprotein E ɛ4 (APOE ɛ4) Allele is Associated with Long Sleep Duration Among Elderly with Cognitive Impairment. J. Alzheimer’s Dis..

[49] Lim A.S.P., Yu L., Kowgier M., Schneider J.A., Buchman A.S., Bennett D.A. (2013). Modification of the Relationship of the Apolipoprotein E ε4 Allele to the Risk of Alzheimer Disease and Neurofibrillary Tangle Density by Sleep. JAMA Neurol..

[50] Drogos L.L., Gill S.J., Tyndall A.V., Raneri J.K., Parboosingh J.S., Naef A., Guild K.D., Eskes G., Hanly P.J., Poulin M.J. (2016). Evidence of association between sleep quality and APOE ε4 in healthy older adults: A pilot study. Neurology.

[51] Zhou L., Gao Q., Nie M., Gu J.-L., Hao W., Wang L., Cao J.-M. (2016). Degeneration and energy shortage in the suprachiasmatic nucleus underlies the circadian rhythm disturbance in ApoE−/− mice: Implications for Alzheimer’s disease. Sci. Rep..

[52] Melkani G.C., Panda S. (2017). Time-restricted feeding for prevention and treatment of cardiometabolic disorders. J. Physiol..

[53] Hatori M., Vollmers C., Zarrinpar A., DiTacchio L., Bushong E.A., Gill S., Leblanc M., Chaix A., Joens M., Fitzpatrick J.A.J. (2012). Time-Restricted Feeding without Reducing Caloric Intake Prevents Metabolic Diseases in Mice Fed a High-Fat Diet. Cell Metab..

[54] Villanueva J.E., Livelo C., Trujillo A.S., Chandran S., Woodworth B., Andrade L., Le H.D., Manor U., Panda S., Melkani G.C. (2019). Time-restricted feeding restores muscle function in Drosophila models of obesity and circadian-rhythm disruption. Nat. Commun..

[55] Chaix A., Manoogian E.N.C., Melkani G.C., Panda S. (2019). Time-Restricted Eating to Prevent and Manage Chronic Metabolic Diseases. Annu. Rev. Nutr..

[56] Sundaram S., Yan L. (2016). Time-restricted feeding reduces adiposity in mice fed a high-fat diet. Nutr. Res..

[57] Duncan M.J., Smith J.T., Narbaiza J., Mueez F., Bustle L.B., Qureshi S., Fieseler C., Legan S.J. (2016). Restricting feeding to the active phase in middle-aged mice attenuates adverse metabolic effects of a high-fat diet. Physiol. Behav..

[58] Tsimakouridze E.V., Alibhai F.J., Martino T.A. (2015). Therapeutic applications of circadian rhythms for the cardiovascular system. Front Pharmacol..

[59] Roth J.R., Varshney S., de Moraes R.C.M., Melkani G.C. (2023). Circadian-mediated regulation of cardiometabolic disorders and aging with time-restricted feeding. Obesity.

[60] Sutton E.F., Beyl R., Early K.S., Cefalu W.T., Ravussin E., Peterson C.M. (2018). Early Time-Restricted Feeding Improves Insulin Sensitivity, Blood Pressure, and Oxidative Stress Even without Weight Loss in Men with Prediabetes. Cell Metab..

[61] Gabel K., Hoddy K.K., Haggerty N., Song J., Kroeger C.M., Trepanowski J.F., Panda S., Varady K.A. (2018). Effects of 8-hour time restricted feeding on body weight and metabolic disease risk factors in obese adults: A pilot study. Nutr. Healthy Aging.

[62] King M.W., Chen Y., Musiek E.S. (2023). Time-restricted feeding and Alzheimer’s disease: You are when you eat. Trends Mol. Med..

[63] Whittaker D.S., Akhmetova L., Carlin D., Romero H., Welsh D.K., Colwell C.S., Desplats P. (2023). Circadian modulation by time-restricted feeding rescues brain pathology and improves memory in mouse models of Alzheimer’s disease. Cell Metab..

[64] Gill S., Le H.D., Melkani G.C., Panda S. (2015). Time-restricted feeding attenuates age-related cardiac decline in Drosophila. Science.

[65] Piazza N., Wessells R.J. (2011). Drosophila models of cardiac disease. Prog. Mol. Biol. Transl. Sci..

[66] Ruiz M., Sanchez D., Canal I., Acebes A., Ganfornina M.D. (2011). Sex-dependent modulation of longevity by two Drosophila homologues of human Apolipoprotein D, GLaz and NLaz. Exp. Gerontol..

[67] Livelo C., Guo Y., Abou Daya F., Rajasekaran V., Varshney S., Le H.D., Barnes S., Panda S., Melkani G.C. (2023). Time-restricted feeding promotes muscle function through purine cycle and AMPK signaling in Drosophila obesity models. Nat. Commun..

[68] Melkani G.C., Trujillo A.S., Ramos R., Bodmer R., Bernstein S.I., Ocorr K. (2013). Huntington’s disease induced cardiac amyloidosis is reversed by modulating protein folding and oxidative stress pathways in the Drosophila heart. PLoS Genet..

[69] Koushika S.P., Lisbin M.J., White K. (1996). ELAV, a Drosophila neuron-specific protein, mediates the generation of an alternatively spliced neural protein isoform. Curr. Biol..

[70] Sanchez D., López-Arias B., Torroja L., Canal I., Wang X., Bastiani M.J., Ganfornina M.D. (2006). Loss of Glial Lazarillo, a Homolog of Apolipoprotein D, Reduces Lifespan and Stress Resistance in Drosophila. Curr. Biol..

[71] Bryantsev A.L., Baker P.W., Lovato T.L., Jaramillo M.S., Cripps R.M. (2012). Differential requirements for Myocyte Enhancer Factor-2 during adult myogenesis in Drosophila. Dev. Biol..

[72] Brand A.H., Perrimon N. (1993). Targeted gene expression as a means of altering cell fates and generating dominant phenotypes. Development.

[73] Freeman M.R. (2015). Drosophila Central Nervous System Glia. Cold Spring Harb. Perspect. Biol..

[74] Ou J., Gao Z., Song L., Ho M.S. (2016). Analysis of Glial Distribution in Drosophila Adult Brains. Neurosci. Bull..

[75] McFie P.J., Banman S.L., Kary S., Stone S.J. (2011). Murine Diacylglycerol Acyltransferase-2 (DGAT2) Can Catalyze Triacylglycerol Synthesis and Promote Lipid Droplet Formation Independent of Its Localization to the Endoplasmic Reticulum. J. Biol. Chem..

[76] Kinney J.W., Bemiller S.M., Murtishaw A.S., Leisgang A.M., Salazar A.M., Lamb B.T. (2018). Inflammation as a central mechanism in Alzheimer’s disease. Alzheimer’s Dement. Transl. Res. Clin. Interv..

[77] Weaver L.N., Ma T., Drummond-Barbosa D. (2020). Analysis of Gal4 Expression Patterns in Adult Drosophila Females. G3.

[78] Yenilmez B., Wetoska N., Kelly M., Echeverria D., Min K., Lifshitz L., Alterman J.F., Hassler M.R., Hildebrand S., DiMarzio C. (2022). An RNAi therapeutic targeting hepatic DGAT2 in a genetically obese mouse model of nonalcoholic steatohepatitis. Mol. Ther..

[79] Rong S., Xia M., Vale G., Wang S., Kim C.-W., Li S., McDonald J.G., Radhakrishnan A., Horton J.D. (2024). DGAT2 inhibition blocks SREBP-1 cleavage and improves hepatic steatosis by increasing phosphatidylethanolamine in the ER. Cell Metab..

[80] Hamilton L.K., Moquin-Beaudry G., Mangahas C.L., Pratesi F., Aubin M., Aumont A., Joppé S.E., Légiot A., Vachon A., Plourde M. (2022). Stearoyl-CoA Desaturase inhibition reverses immune, synaptic and cognitive impairments in an Alzheimer’s disease mouse model. Nat. Commun..

[81] Astarita G., Jung K.-M., Vasilevko V., DiPatrizio N.V., Martin S.K., Cribbs D.H., Head E., Cotman C.W., Piomelli D. (2011). Elevated Stearoyl-CoA Desaturase in Brains of Patients with Alzheimer’s Disease. PLoS ONE.

[82] Wood P.L., Medicherla S., Sheikh N., Terry B., Phillipps A., Kaye J.A., Quinn J.F., Woltjer R.L. (2015). Targeted Lipidomics of Fontal Cortex and Plasma Diacylglycerols (DAG) in Mild Cognitive Impairment and Alzheimer’s Disease: Validation of DAG Accumulation Early in the Pathophysiology of Alzheimer’s Disease. J. Alzheimer’s Dis..

[83] Man W.C., Miyazaki M., Chu K., Ntambi J. (2006). Colocalization of SCD1 and DGAT2: Implying preference for endogenous monounsaturated fatty acids in triglyceride synthesis. J. Lipid Res..

[84] van Dyck C.H., Swanson C.J., Aisen P., Bateman R.J., Chen C., Gee M., Kanekiyo M., Li D., Reyderman L., Cohen S. (2023). Lecanemab in Early Alzheimer’s Disease. N. Engl. J. Med..

[85] Cummings J., Apostolova L., Rabinovici G.D., Atri A., Aisen P., Greenberg S., Hendrix S., Selkoe D., Weiner M., Petersen R.C. (2023). Lecanemab: Appropriate Use Recommendations. J. Prev. Alzheimer’s Dis..

[86] Arbanas J.C., Damberg C.L., Leng M., Harawa N., Sarkisian C.A., Landon B.E., Mafi J.N. (2023). Estimated Annual Spending on Lecanemab and Its Ancillary Costs in the US Medicare Program. JAMA Int. Med..

[87] Whittaker D.S., Akhmetova L., Colwell C.S., Desplats P. (2021). A time-restricted feeding intervention reduces alterations in circadian behaviors and pathology in a mouse model of Alzheimer’s disease. Alzheimer’s Dement..

[88] Hardie D.G., Pan D.A. (2002). Regulation of fatty acid synthesis and oxidation by the AMP-activated protein kinase. Biochem. Soc. Trans..

