# Supplementary material for: Apolipoprotein E Induces Lipid Accumulation Through Dgat2 That Is Prevented with Time-Restricted Feeding in Drosophila
Source: Genes (Basel). 2024 Oct 25;15(11):1376. doi: 10.3390/genes15111376 (PMC11594465; doi:10.3390/genes15111376)
Supplement: Supplementary file 1 [file genes-15-01376-s001.zip › Supplementary Document S1.pdf]

Apolipoprotein E induces lipid accumulation through Dgat2 that is prevented with time-restricted feeding in *Drosophila*

Supplementary Figures

Ruan Carlos Macedo de Moraes[1,2¶], Jonathan R. Roth[1,3¶], Hailey Mao[1], Savannah R. Crawley[1], Brittney P. Xu[1], John C. Watson[1], Girish C. Melkani[1,4*]

[1]Department of Pathology, Division of Molecular and Cellular Pathology, Heersink School of Medicine, University of Alabama at Birmingham, Birmingham, AL 35294, USA.

[2]Department of Psychiatry and Behavioral Neurobiology, Heersink School of Medicine, University of Alabama at Birmingham, Birmingham, AL 35294, USA.

[3]Department of Neurobiology, Heersink School of Medicine, University of Alabama at Birmingham, Birmingham, AL 35294, USA.

[4]UAB Nathan Shock Center, 1300 University Boulevard Birmingham, AL 35294, USA.

[*]Corresponding Address: Department of Pathology, Division of Molecular and Cellular Pathology, Heersink School of Medicine, University of Alabama at Birmingham, AL 35294, USA. Tel.: 1-205-996-0591; Fax: 1-205-934-7447; E-mail: girishmelkani@uabmc.edu (GCM)

¶ These authors contributed equally to this work

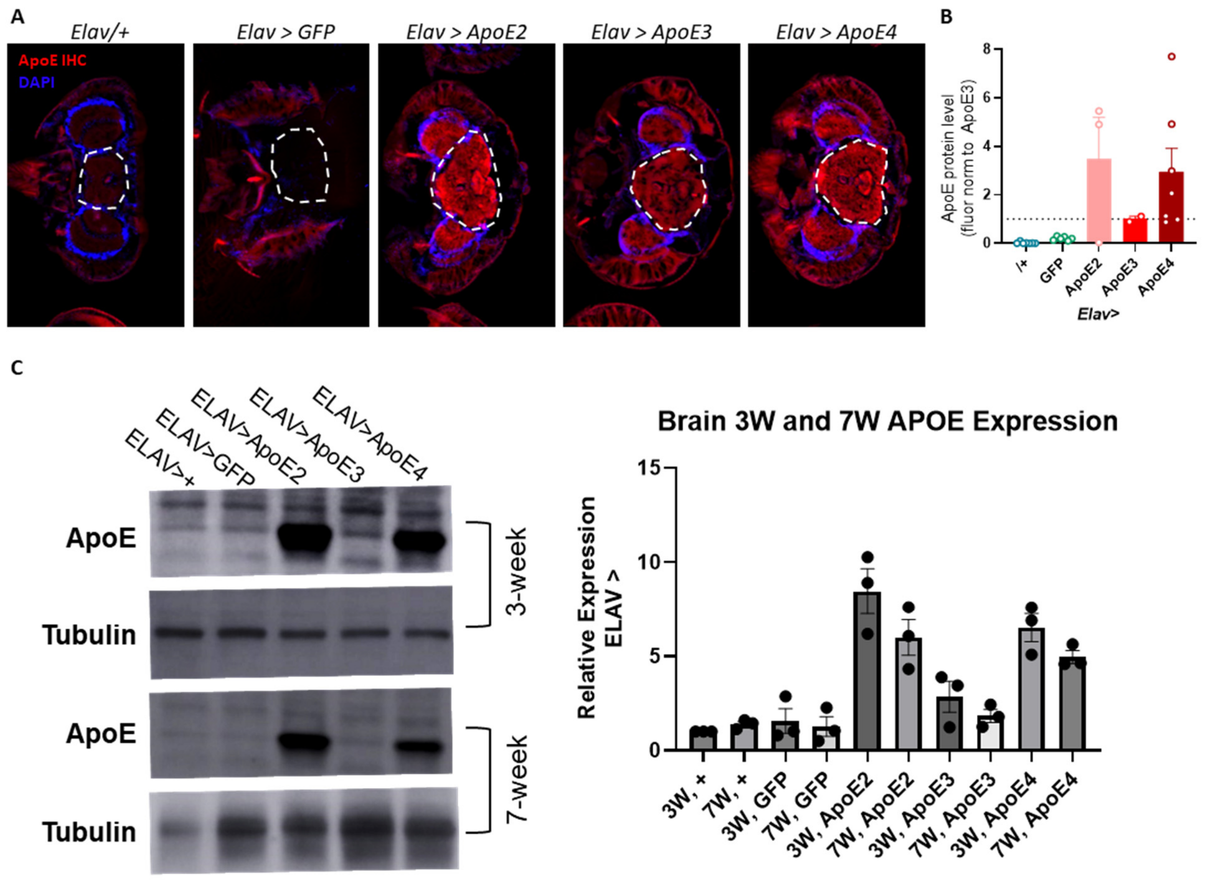

Supplementary Figure S1: ApoE expression in the brain. A: Representative images of 3 week old female fly heads immunostained for ApoE demonstrates that Elav > ApoE2/3/4 flies express ApoE protein in the brain (white circle). B: Quantification of ApoE immunohistochemistry fluorescence in the brain (N = 2-7 flies per group). C: Western blot of ApoE expression in both 3 week and 7 week old fly heads show increased expression in ApoE for all three alleles and no change with age.

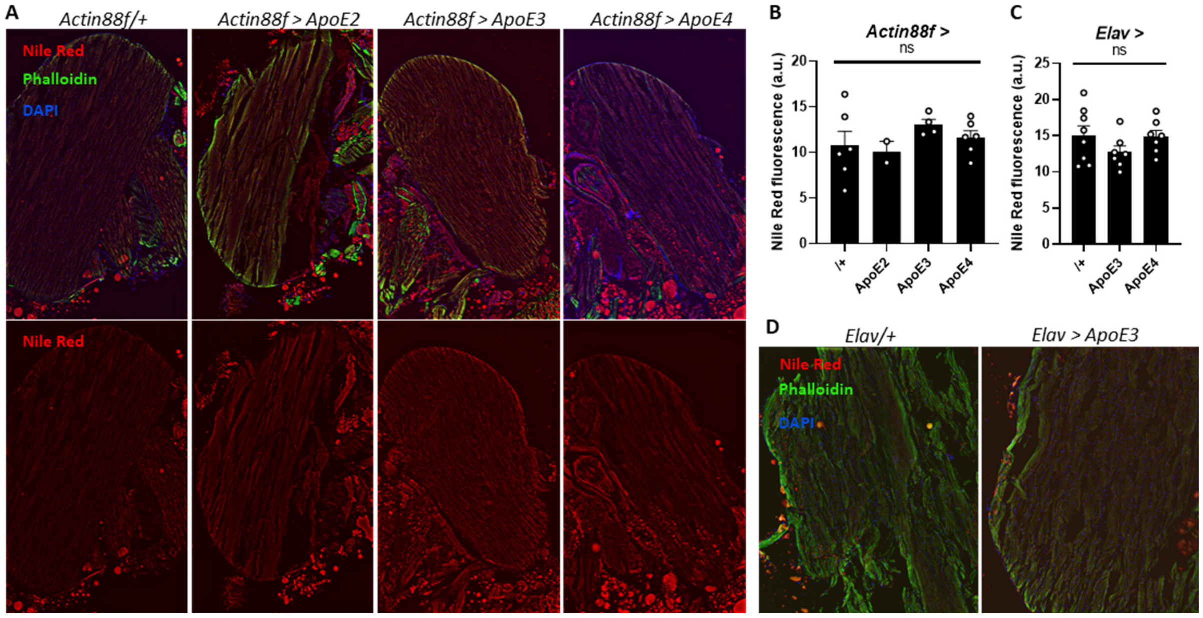

Supplementary Figure S2: ApoE does not induce lipid accumulation in flight muscles. A: Representative images of flight muscles stained for lipids (Nile Red) and actin (Phalloidin) show no increase in lipid accumulation in flies expressing ApoE2/3/4 in flight muscles with the *Actin88f-Gal4* driver. B: Quantification of Nile Red in flight muscles show no changes in lipid levels between genotypes when ApoE is expressed in flight muscles (ANOVA $F(3,14) = 0.82$, $p = 0.51$, $n = 2$-6 flies per group). C: Quantificaiton of Nile Red in flight muscles show no changes in lipid levels between genotypes when ApoE is expressed in neurons (ANOVA $F(2,19) = 1.42$, $p = 0.27$, $n = 7$-8 flies per group). D: Representative images show no changes in lipid levels in flight muscles when ApoE is expressed in neurons with the *Elav-Gal4* driver.

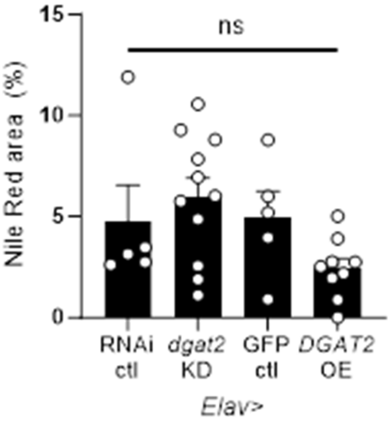

Supplementary Figure S3: Manipulating Dgat2 level in neurons does not significantly alter lipid accumulation in the brain. A: There is no significant difference in quantification of lipid accumulation in the brain after overexpressing *DGAT2* of knocking down *dgat2* compared to GFP and RNAi controls (ANOVA $F(3,26) = 2.6$, $p = 0.08$, $N = 5$-11 flies per group).

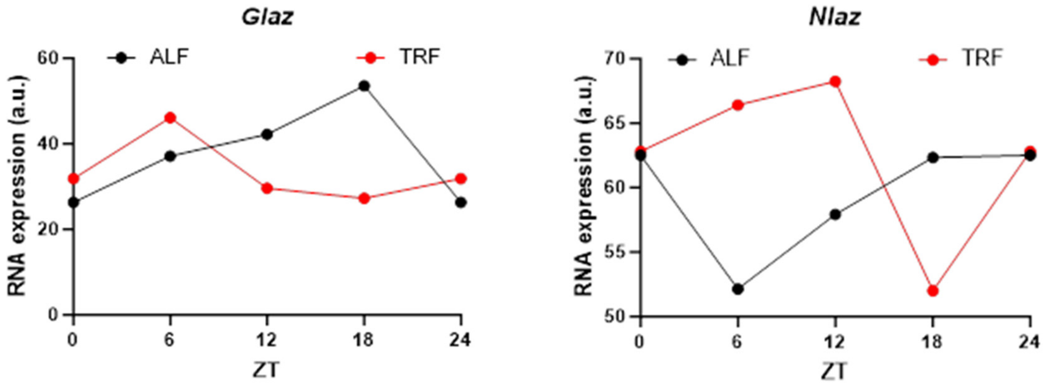

Supplementary Figure S4: TRF changes the peak expression of brain apoliproteins *Glaz* and *Nlaz*. After two weeks of TRF, 3 week old fly heads were isolated every six hours and RNAseq was performed to assess rhythmic expression of apolipoproteins Glaz and Nlas. TRF shifted peak expression of *Glaz* from ZT18 to ZT6. TRF shifted *Nlaz* peak expression from ZT 0/24 to ZT12. Data obtained from our prior publication (Gill 2015).

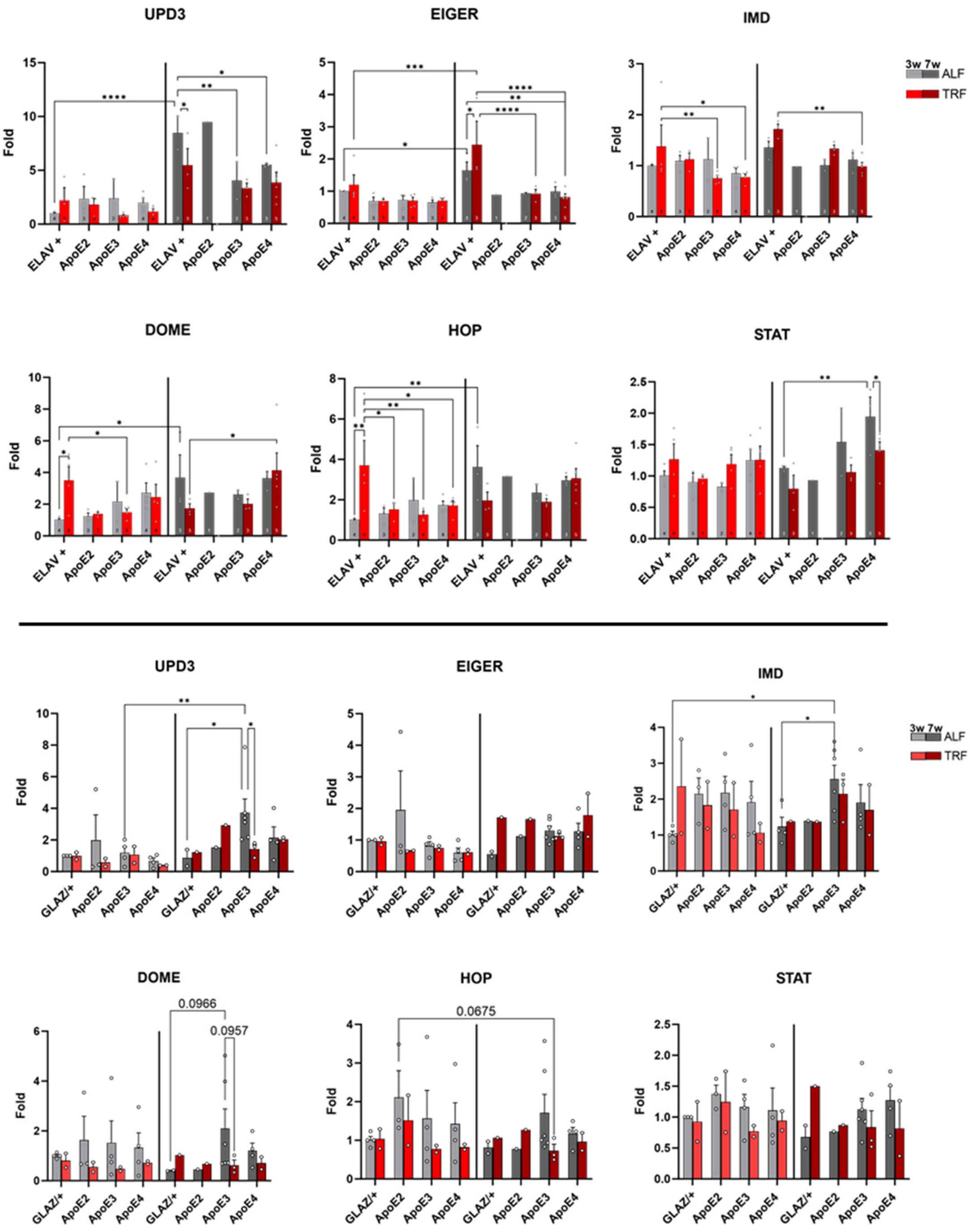

Supplementary Figure S5: Expression of inflammation-related genes with aging and TRF. Effects of time-restricted feeding (TRF) on the expression of genes associated with infflamation in adult (3 weeks old) and old-adult (7 weeks old) flies using a pan-neuronal (ELAV) and pan-glial (GLaz) driver, show that there is no specific effect of ApoE in the gene expression of inflammation-related genes. Mixed-effects analysis was conducted, and post-hoc Fisher LSD test (* p < 0.05; ** p < 0.01; *** p < 0.001; **** p < 0.0001).
